# Non-linear relationship between baseline fasting blood glucose and mortality in peritoneal dialysis patients, a retrospective cohort study

**DOI:** 10.3389/fmed.2024.1325914

**Published:** 2024-02-16

**Authors:** Xiang Li, Chengjuan Fan, Chen Wang, Yiming Zhang, Lingling Niu

**Affiliations:** ^1^Department of Nephrology, Affiliated Hospital of Jining Medical University, Jining, China; ^2^Department of Gastroenterology, Affiliated Hospital of Jining Medical University, Jining, China

**Keywords:** fasting blood glucose, mortality, peritoneal dialysis, cox regression, retrospective study

## Abstract

**Background:**

The relationship between baseline fasting blood glucose (bFBG) and mortality in peritoneal dialysis (PD) patients has been the subject of debate, with limited exploration of the non-linear relationship between bFBG and death in these patients.

**Methods:**

This retrospective study categorized patients into four groups based on their bFBG using quartiles. Baseline clinical data at the initiation of dialysis were compared. Survival curves were plotted, and subgroup analyses were stratified by relevant covariates. To address the non-linear relationship, curve fitting and a threshold effect analysis were performed.

**Results:**

The study included 379 PD patients with a median follow-up of 41.8 (22.6, 60.1) months. The COX proportional hazards model showed an association between bFBG and the risk of death after adjusting for confounding factors [hazard ratio (*HR*): 1.22, 95% CI: 1.05−1.41, *P* = 0.009]. Stratified analyses indicated a stable correlation between bFBG and mortality. The Kaplan-Meier curve analysis revealed significant differences in survival rates among different groups based on bFBG levels (*P* < 0.01). The curve fitting analysis revealed a U-shaped relationship between bFBG and mortality, with an inflection point at approximately 5.1 mmol/L.

**Conclusion:**

Our study has demonstrated a non-linear relationship between bFBG and mortality in PD patients. Additionally, we have found that the optimal bFBG value associated with the lowest risk of mortality is approximately 5.1 mmol/L.

## 1 Introduction

As a home-based therapy, PD offers several inherent advantages, including the preservation of residual renal function, maintenance of hemodynamics, improvement in the quality of life, and cost savings (1). Additionally, studies suggest that PD demonstrates a comparable, or even superior, survival rate compared to in-center hemodialysis (HD) (2, 3).

However, due to the common use of glucose as an osmotic agent in PD solutions, patients undergoing PD have a higher risk of experiencing abnormal glucose metabolism and developing new-onset diabetes compared to those receiving HD (4–6). Although glucose is inexpensive, effective, easily metabolized, and contributes to daily energy intake, the absorption of glucose from peritoneal dialysate can result in hyperglycemia, dyslipidemia, and weight gain. These complications further exacerbate the presence of metabolic syndrome and increase the risk of cardiovascular disease in PD patients (7). Poor glycemic control is typically associated with unfavorable outcomes in both non-dialyzed patients and patients undergoing dialysis, as indicated by research from various countries and regions (8–10). However, another study did not observe an association between glucose control, as assessed by baseline and follow-up values of glycated hemoglobin, and survival of diabetes patients undergoing PD (11).

However, the above-mentioned studies did not conduct detailed research on the bFBG levels before the start of PD treatment. Furthermore, most studies only focused on the correlation between high blood glucose levels and mortality rates, with relatively few studies on the association between low blood glucose levels and mortality rates. Our retrospective cohort study aims to observe the impact of bFBG levels before PD treatment on mortality rates and attempts to identify the optimal target value for blood glucose control.

## 2 Materials and methods

### 2.1 Data collection

Patient data, including demographic information, medical history, and laboratory results, were collected from the hospital’s electronic medical records system. The bFBG levels were recorded at the start of PD treatment. Demographic variables such as age, gender, and smoking status were also recorded.

### 2.2 Patients

All patients (*n* = 473) with end-stage renal disease (ESRD) at the nephrology division of Jining Medical University Affiliated Hospital who chose to undergo regular continuous ambulatory peritoneal dialysis (CAPD) were included in the study. The enrollment period was from January 1, 2013, to December 31, 2022. All patients were followed up for a minimum of 6 months until the study concluded on June 30, 2023. We established a patient cohort that met the following inclusion criteria: (1) patients who underwent daily PD successfully; (2) adult PD patients aged 18 years or older; (3) PD patients who could attend regular follow-up and had complete clinical data. Patients who underwent hemodialysis or kidney transplant were considered lost to follow-up and were excluded from the study.

### 2.3 Evaluation of bFBG

The venous fasting blood glucose (FBG) tested prior to the initiation of regular PD treatment served as the bFBG.

### 2.4 Evaluation of other covariates

Prognostic variables for mortality were gathered based on clinical experience and previous studies. These variables encompassed Age, Sex, Smoking status, Diabetes, Hypertension, Antihyperglycemic medications (AHM, including insulin or oral hypoglycemic agents), Antihypertensive drugs (AHD), White Blood Cell Count, Hemoglobin, Platelet Count, Mean Corpuscular Volume (MCV), Alanine Aminotransferase (ALT), Aspartate Aminotransferase, Alkaline Phosphatase, Total Protein (TP), Albumin (ALB), Prealbumin, Blood Urea Nitrogen, Creatinine (CR), Uric Acid (UA), Corrected Calcium (cCa), Phosphorus (P), Parathyroid Hormone (PTH), Total Cholesterol, Triglycerides (TG), High-Density Lipoprotein, Low-Density Lipoprotein, Weekly Creatinine Clearance (Wkly Ccr), Weekly Total Kt/V (Wkly Total Kt/V), Renal Weekly Kt/V (RW Kt/V), and PD Weekly Kt/V (PD Wkly Kt/V). Blood samples were collected before the initiation of PD, and the time between blood sampling and specimen analysis was less than 30 min. The results for Wkly Ccr, Wkly Total Kt/V, RW Kt/V, and PD Wkly Kt/V were calculated approximately 1°month after regular PD by obtaining blood samples, 24-hour peritoneal dialysis effluent, and urine samples.

### 2.5 Follow-up data

We conducted the follow-up through specialized outpatient clinics. Follow-up was conducted for all patients until death, or the end of the follow-up period (June 30, 2023). All-cause mortality analysis defined death as the endpoint event, with time defined as the number of months from the patient’s entry into PD to death or the end of the follow-up.

### 2.6 Statistical analysis

A descriptive analysis was conducted for all study participants. Categorical variables were presented as frequencies and percentages. For continuous variables, we reported the mean and standard deviation (SD) when they followed a normal distribution, and the median and interquartile range (IQR) for those with skewed distributions. We employed the chi-square test, one-way ANOVA, and Kruskal-Wallis test to compare categorical variables, normally distributed continuous variables, and non-normally distributed continuous variables, respectively, as required for the academic paper. The baseline characteristics were presented based on quartile grouping of bFBG (Q1, bFBG < 4.6 mmol/L; Q2, 4.6 mmol/L ≤ bFBG < 5.18 mmol/L; Q3, 5.18 mmol/L ≤ bFBG < 5.73 mmol/L; Q4, bFBG ≥ 5.73 mmol/L).

Multivariable COX regression analysis was adopted to assess the independent association between bFBG and mortality. We included variables with a *P*-value less than 0.1 from the univariate COX regression analysis, as well as those identified based on a thorough review of existing literature and clinical experience, in the multivariate COX regression for confounding factor correction.

Survival was assessed by constructing Kaplan-Meier survival curves based on bFBG quartiles and evaluated using the log-rank test.

In order to further investigate the relationship between bFBG and mortality, we will utilize the Locally Estimated Scatterplot Smoothing (LOESS) method for smooth curve fitting. Based on curve fitting using restricted cubic splines, we conducted a two-piecewise linear regression model to identify threshold effects if a non-linear correlation was observed. The threshold levels of bFBG were determined using a recursive method, resulting in a maximum likelihood model.

A sensitivity analysis was conducted to validate the robustness of the data analysis. First, bFBG was transformed into a categorical variable. Secondly, to assess the impact of potential sources of bias on the results, we also conducted COX multivariable regression using the raw data before imputation.

Stratified and interaction analyses were conducted based on Sex (male or female), Age (<65 or ≥65 years), Smoking status (yes or no), and Diabetes (yes or no).

The percentages of covariates with missing data were below 30% for all analyses. We used multiple imputations to impute the missing values of the covariates. Our study utilized multiple imputation procedures for five imputations. To enhance the robustness of the results, we combined the datasets from these five imputations and calculated the average. All variables were subjected to multicollinearity diagnostics.

All analyses were conducted using the R statistical software (version 4.0.3; The R Foundation)^1^ and Free Statistics Software (version 1.8). In our statistical analysis and visualization, we utilized various R packages. Specifically, we applied the “survival” package for survival analysis, “survminer” for visualizing survival analysis, “rms” for fitting curves and constructing models, “segmented” for segmented regression analysis, “ggplot2” for creating charts, “mice” for multiple imputation, and “car” for statistical analysis and diagnostic checks in regression models. Statistical significance was assessed using a two-tailed test with a significance threshold set at *P* < 0.05.

## 3 Results

### 3.1 Population

**FIGURE 1:**
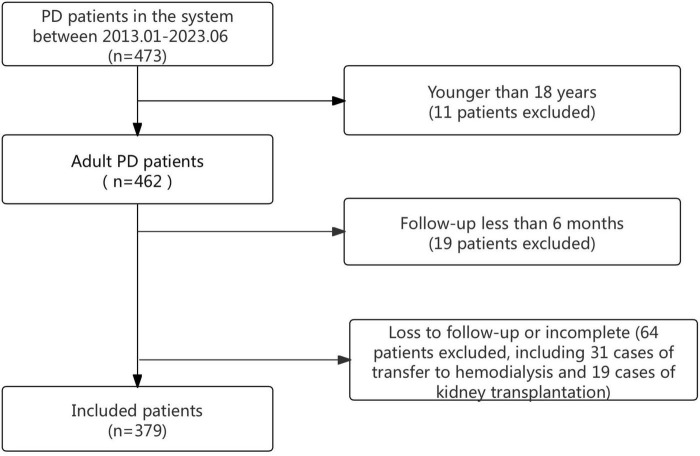
The flow chart of the study.

In total, 473 PD patients were identified from the electronic medical record system. Among them, 11 cases younger than 18 years old were excluded, 19 cases with a follow-up period of less than 6 months were excluded, and 64 cases, including 31 cases of transfer to hemodialysis and 19 cases of kidney transplantation, with loss to follow-up or incomplete case data were excluded. A total of 379 cases were included in the cohort (Figure 1 shows a flow chart).

### 3.2 Baseline characteristics

The basic demographic characteristics of the selected patients are summarized in Table 1, stratified by bFBG quartile. Overall, the participants had a median follow-up time of 41.8 (22.6, 60.1) months, with an average age of 48.2 ± 13.6 years, and approximately 63.9% were male. The mortality rate was 11.9% (45 out of 379). We observed significant differences in Age, Smoking status, Diabetes, Hypertension, AHM, ALT, TP, ALB, PAB, CR, cCa, PTH, and TG among the different groups.

**TABLE 1:** Patient demographics and baseline characteristics.

Characteristic	bFBG					*P*
	**Total (*n* = 379)**	**Q1**, ***N* = 82**	**Q2, *N* = 107**	**Q3, *N* = 95**	**Q4, *N* = 95**	
Male *n* (%)	239 (63.1)	50 (61)	68 (63.6)	60 (63.2)	61 (64.2)	0.975
Age (year)	48.2 ± 13.6	46.0 ± 13.4	46.3 ± 13.5	46.0 ± 13.1	54.3 ± 12.5	<0.001
Smoking *n* (%)	78 (20.6)	15 (18.3)	13 (12.1)	21 (22.1)	29 (30.5)	0.013
Diabetes *n* (%)	62 (16.4)	4 (4.9)	6 (5.6)	2 (2.1)	50 (52.6)	<0.001
Hypertension, *n* (%)	315 (83.1)	62 (75.6)	86 (80.4)	86 (90.5)	81 (85.3)	0.048
AHM (yes)	35 (9.2)	8 (9.8)	18 (16.8)	3 (3.2)	6 (6.3)	0.006
AHD (yes)	278 (73.4)	57 (69.5)	75 (70.1)	76 (80)	70 (73.7)	0.34
WBC (×10^9)	6.4 ± 2.2	6.3 ± 1.9	6.5 ± 2.6	6.4 ± 2.0	6.4 ± 2.0	0.916
Hb (g/L)	98.4 ± 19.2	99.7 ± 21.3	96.6 ± 20.5	99.1 ± 16.7	98.3 ± 18.4	0.695
PLA (×10^9)	200.1 ± 69.6	189.3 ± 69.7	201.0 ± 76.6	207.1 ± 66.6	201.2 ± 63.8	0.394
MCV (fL)	91.5 ± 5.3	92.6 ± 5.5	91.7 ± 4.7	90.6 ± 5.3	91.4 ± 5.4	0.091
ALT (U/L)	17.0 (10.4, 38.1)	20.6 (13.0, 55.8)	13.6 (9.5, 29.4)	15.5 (10.1, 31.5)	17.0 (10.2, 30.5)	0.019
AST (U/L)	17.0 (12.0, 23.0)	17.0 (12.0, 23.1)	16.0 (11.5, 22.0)	17.0 (12.5, 22.0)	17.0 (13.0, 24.0)	0.366
ALP (U/L)	82.0 (65.0, 108.0)	76.0 (63.2, 101.5)	86.0 (66.5, 129.5)	81.0 (61.5, 98.5)	83.0 (68.8, 122.0)	0.153
TP (g/L)	61.7 ± 8.0	58.0 ± 7.5	61.8 ± 7.8	64.8 ± 7.8	61.6 ± 7.7	<0.001
ALB (g/L)	36.5 ± 5.8	34.5 ± 5.4	36.8 ± 5.5	38.7 ± 5.1	35.8 ± 6.3	<0.001
PAB (g/L)	296.0 (37.9, 390.0)	46.3 (34.0, 327.0)	303.0 (39.4, 387.5)	348.0 (41.5, 424.5)	306.0 (41.9, 366.5)	<0.001
BUN (mmol/L)	25.7 ± 10.0	27.0 ± 9.7	26.3 ± 10.5	24.7 ± 9.4	25.0 ± 10.4	0.356
CR (umol/L)	733.1 (587.0, 966.1)	794.3 (626.8, 984.4)	783.0 (614.5, 994.4)	729.0 (585.5, 951.0)	663.0 (534.3, 893.2)	0.031
UA (umol/L)	398.8 ± 112.8	391.1 ± 106.1	406.0 ± 117.0	398.4 ± 99.0	397.8 ± 126.9	0.842
cCa (mmol/L)	2.2 ± 0.3	2.1 ± 0.3	2.2 ± 0.2	2.2 ± 0.3	2.3 ± 0.3	0.011
P (mmol/L)	1.8 ± 0.6	1.8 ± 0.5	1.8 ± 0.6	1.7 ± 0.5	1.7 ± 0.6	0.256
PTH (pg/mL)	269.9 (158.7, 447.0)	270.7 (171.5, 431.1)	336.0 (190.5, 545.3)	266.1 (160.2, 428.8)	234.7 (123.0, 350.7)	0.002
TC (mmol/L)	4.6 ± 1.2	4.2 ± 1.1	4.5 ± 1.1	4.7 ± 1.3	4.7 ± 1.3	0.037
TG (mmol/L)	1.3 (0.9, 1.8)	1.1 (0.8, 1.6)	1.3 (0.9, 1.7)	1.4 (1.0, 1.7)	1.4 (1.0, 2.3)	0.005
HDL (mmol/L)	1.2 ± 0.4	1.1 ± 0.4	1.2 ± 0.4	1.2 ± 0.4	1.2 ± 0.4	0.633
LDL (mmol/L)	2.6 ± 0.9	2.4 ± 0.8	2.6 ± 0.8	2.7 ± 0.8	2.7 ± 1.0	0.053
Wkly Ccr (ml/min)	78.0 ± 28.2	74.8 ± 24.4	78.4 ± 25.2	79.9 ± 36.8	78.3 ± 25.1	0.717
Wkly Total Kt/V	2.0 (1.7, 2.4)	2.1 (1.8, 2.4)	2.0 (1.8, 2.6)	2.0 (1.7, 2.5)	2.0 (1.7, 2.4)	0.668
PD Wkly Kt/V	0.6 (0.4, 0.9)	0.6 (0.3, 0.9)	0.7 (0.4, 0.9)	0.6 (0.4, 0.9)	0.6 (0.4, 1.0)	0.72
RW Kt/V	1.4 (1.2, 1.7)	1.4 (1.2, 1.7)	1.4 (1.2, 1.7)	1.4 (1.2, 1.6)	1.4 (1.2, 1.7)	0.678
Follow-up period (month)	41.8 (22.6, 60.1)	47.7 (27.1, 67.9)	42.1 (23.3, 60.3)	41.1 (22.3, 56.7)	34.8 (22.1, 50.7)	0.141

Data presented are mean ± SD, median (IQR), or *N* (%). AHM, Antihyperglycemic medications; AHD, antihypertensive drugs; WBC, White Blood Cell Count; Hb, Hemoglobin; PTT, Platelet Count; MCV, Mean Corpuscular Volume; ALT, Alanine Aminotransferase; AST, Aspartate Aminotransferase; ALP, Alkaline Phosphatase; TP, Total Protein; ALB, Albumin; PAB, Prealbumin; BUN, Blood Urea Nitrogen; CR, Creatinine; UA, Uric Acid; cCa, Corrected Calcium; PTH, Phosphorus Parathyroid Hormone; TC, Total Cholesterol; TG, Triglycerides; HDL, High-Density Lipoprotein; LDL, Low-Density Lipoprotein; Wkly Ccr, Weekly creatinine clearance; Wkly Total Kt/V, Weekly Total Kt/V; RW Kt/V, Renal Weekly Kt/V; PD Wkly Kt/V, Peritoneal Dialysis Weekly Kt/V.

### 3.3 Multivariable COX regression analysis

By selecting variables with a *P*-value less than 0.1 in the univariate COX regression analysis and based on a comprehensive review of existing literature and clinical experience, we identified variables, including Age, Sex, Diabetes, Smoking status, MCV, UA, ALB, Cr, and P, as confounding variables for correction in the multivariable COX regression model. For detailed results of the univariate COX regression analysis, please refer to Supplementary Table 1. We constructed three models to analyze the independent impact of bFBG on mortality using a multivariate COX regression model (Table 2). The results indicated an association between bFBG and the risk of death after adjusting for confounding factors (*HR* 1.22, 95% *CI*: 1.05−1.41, *P* = 0.009). Notably, we found that the *HRs* remained consistent across all three models (*P* < 0.05). For additional sensitivity analysis, the continuous variable bFBG was discretized into a categorical variable based on quartiles, with Q1 serving as the baseline reference. The HRs for Q2 were found to be lower than those for Q1 (*HR*: 0.73, 95% *CI* 0.21−0.86, *P* = 0.027). Conversely, the *HR*s for Q3 and Q4 were higher than those for Q1. Comparable findings were obtained in the analysis of the original data before imputation (Supplementary Table 2).

**TABLE 2:** Association between initial bFBG and mortality in different models.

Variable	Model 1			Model 2			Model 3		
	***HR* ^1^**	***95%CI^1^***	***P***	***HR* ^1^**	***95%CI^1^***	***P***	***HR* ^1^**	***95%CI^1^***	***P***
bFBG	1.36	1.24, 1.49	<0.001	1.25	1.13, 1.39	<0.001	1.22	1.05, 1.41	0.009
**bFBG**									
Q1	1 (Ref)			1 (Ref)			1 (Ref)		
Q2	0.62	0.2, 0.96	0.416	0.6	0.3, 0.9	0.038	0.73	0.21, 0.86	0.027
Q3	1.52	0.58, 3.99	0.4	1.37	0.52, 3.63	0.523	2.58	0.80, 3.29	0.112
Q4	3.66	1.57, 8.55	0.003	2.39	1,5.71	0.05	1.85	1.56, 6.07	0.012

^1^*HR*, Hazard Ratio; *CI*, confidence interval. Model 1 not adjusted. Model 2 adjusted for Age and Sex. Model 3 adjusted for model 2 plus Diabetes, Smoking status, MCV, UA, ALB, Cr, P.

### 3.4 Kaplan–Meier curves

**FIGURE 2:**
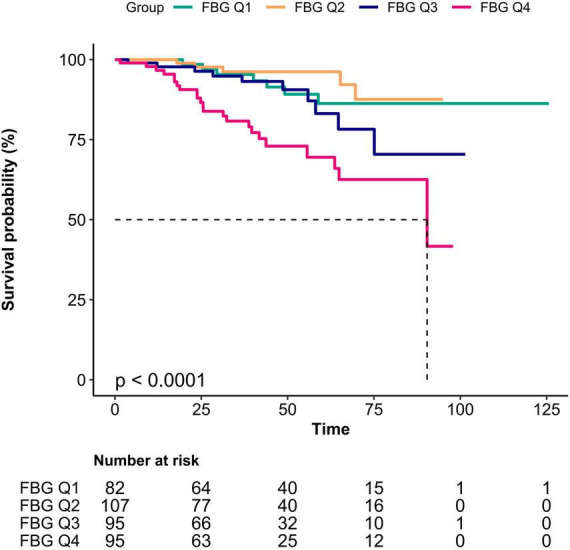
Kaplan-Meier survival curve for mortality according to bFBG.

The Kaplan-Meier curve showed that the overall survival in Q2 was higher than that in Q1, while the survival in Q4 was lower than that in the other three quartiles (log-rank test: *P* < 0.0001; Figure 2).

### 3.5 Subgroup analyses

**FIGURE 3:**
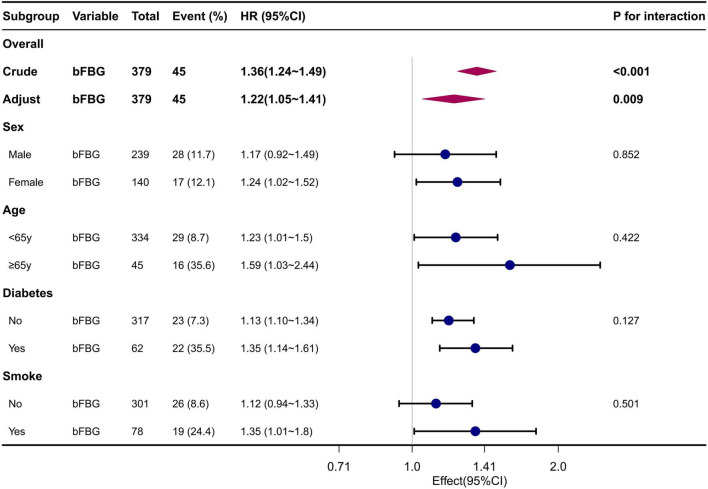
Forest plot for subgroup analysis of the association between FBG and mortality. Adjusted for Sex, Age, Diabetes, Smoking status, MCV, UA, ALB, Cr, P.

Subgroup analyses revealed no significant interaction in the subgroup analysis. This is presented in the forest plot, with all *P*-values for interaction being greater than 0.05 (Figure 3).

### 3.6 The non-linear relationship between bFBG and mortality

**FIGURE 4:**
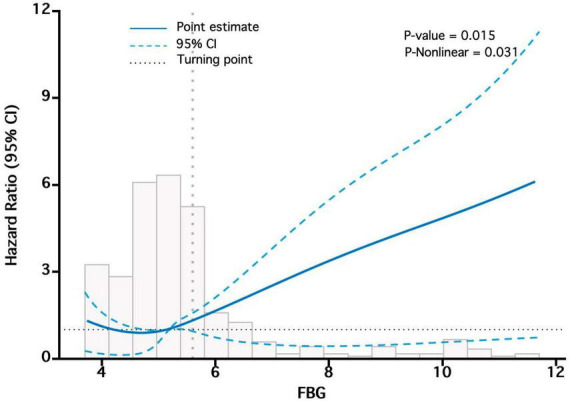
The “U-shaped relationship” between bFBG and mortality. Adjusted for Sex, Age, Diabetes, Smoking status, MCV, UA, ALB, Cr, P.

We observed a non-linear correlation between bFBG and mortality through the use of multivariate COX regression model and smooth curve fitting (Figure 4). The data were fitted to a piecewise multivariate COX regression model, which allowed us to capture two different slopes. In our study, the log-likelihood ratio test yielded a P-value of less than 0.05 (Table 3), indicating the need for a two-piecewise model to accurately depict the relationship between bFBG and mortality. We identified an inflection point at approximately 5.1 mmol/L. On the left side of the inflection point, the effect size was 0.60 (HR: 0.60, 95% CI 0.38−0.94, P = 0.026). On the right side of the inflection point, the effect size was 1.96 (HR: 1.96, 95% CI 1.54−2.51, P < 0.001).

**TABLE 3:** The non-linear relationship between bFBG and Survival.

Threshold of bFBG	*HR*	95% *CI*	*P*
<5.1°mmol/L	0.60	0.38, 0.94	0.026
≥5.1°mmol/L	1.96	1.54, 2.51	<0.001
Likelihood ratio test	−	−	0.008

^1^*HR*, hazard ratio; *CI*^I^, confidence interval. Adjusted for Sex, Age, Diabetes, Smoking status, MCV, UA, ALB, Cr, P.

## 4 Discussion

We discovered that bFBG is an independent factor for all-cause mortality in PD patients. We also conducted multivariable COX regression analysis using the raw data before imputation to assess the impact of potential sources of bias on the results, and similar results were obtained, confirming the robustness of the above findings. The findings from our study align with those of previous research conducted by Rao Kondapally Seshasai et al. (12). However, there are also studies that suggest no association between bFBG and all-cause mortality in the PD population (13–15). These contradictory results may be attributed to different bFBG classification criteria, sample sizes, population characteristics, and incomplete adjustment for potential confounding factors. Therefore, large-scale, multi-center, prospective studies are needed to validate the relationship between baseline FPG and prognosis in PD patients.

When bFBG was converted into a categorical variable, our findings indicated that the HRs of Q2 were lower than that of Q1, suggesting a potential protective effect of bFBG. However, the HRs for Q3 and Q4 were higher than that of Q1, indicating a non-linear association between bFBG and mortality. The survival curve also reveals that the Q2 has the lowest mortality rate, with increased mortality rates in the other three groups. Additionally, curve fitting analysis revealed a U-shaped relationship between bFBG and mortality. This finding is similar to the curve fitting analysis conducted by Chen et al. (16) in a study on the relationship between blood glucose and mortality in a general population in rural China. The piecewise multivariate COX regression shows that when bFBG is less than 5.1 mmol/L, the mortality rate decreases as bFBG increases. However, when bFBG is greater than 5.1 mmol/L, the mortality rate increases with the increase in blood glucose.

Previous studies have shown that high blood glucose at the initiation of PD is associated with an increased risk of mortality in PD patients without pre-existing diabetes (4, 13). Yanyan Shi et al.’ (17) research also demonstrates that new-onset glucose disorders increase the mortality risk in patients undergoing PD. In our study, we found that patients with blood glucose levels between 5.1−6.1°mmol/L, although still within the normal range, may be more prone to developing impaired glucose tolerance or diabetes after undergoing PD treatment, resulting in an increase in mortality rates with higher baseline blood glucose levels.

Recent studies indicate that elevated blood glucose levels can trigger oxidative stress, potentially contributing to the progression and mortality of individuals with chronic kidney disease (18). Diabetic patients often endure a state of chronic inflammation characterized by an increased release of inflammatory factors. This prolonged inflammatory state may result in organ dysfunction, thereby elevating the risk of mortality in PD patients. Furthermore, findings from the study by Soleimanpour et al. (19) suggest that approximately one-third of individuals with high blood glucose levels exhibit ketosis. This higher occurrence of ketosis is associated with an increased risk of death.

Glucose and insulin metabolism are significantly altered in patients with diabetes who have advanced chronic kidney disease (CKD). Several factors contribute to an increased risk of hypoglycemia in these patients, including impaired kidney function in gluconeogenesis, reduced clearance of insulin by the kidneys, compromised insulin degradation due to uremia, enhanced glucose uptake by erythrocytes during HD, impaired responses of counterregulatory hormones (such as cortisol and growth hormone), nutritional deprivation, and variable exposure to oral antihyperglycemic agents and exogenous insulin (20). Therefore, it is common for end-stage renal disease patients to experience hypoglycemia. While the mechanism underlying the relationship between low fasting blood glucose and the risk of all-cause mortality is not entirely clear, studies have demonstrated that hypoglycemia may be linked to reduced energy consumption, leading to poor health and greater vulnerability to diseases (21). Additionally, Ata Mahmoodpoor et al. (22) suggested that there are three possible explanations for the association between hypoglycemia and mortality in critically ill patients. First, they proposed that the severity of hypoglycemia may be correlated with the severity of the underlying illness. Second, they suggested that hypoglycemia may serve as a biomarker for impending death. Third, they hypothesized that hypoglycemia could have a detrimental biological impact on critically ill patients. Consequently, this increases the overall risk of death. In light of our research findings, it is crucial to emphasize the importance of bFBG control prior to PD. The aim should be to maintain glucose levels at around 5.1 mmol/L in order to minimize the mortality rate of ESRD patients after undergoing PD.

The study variables were selected for correction as potential confounding factors based on a thorough review of existing literature, clinical experience, and the univariate COX regression analysis. We utilized a multivariable COX regression model to obtain the study results. It is worth noting that we found MCV to be a risk factor for mortality in PD patients. This finding has been confirmed in hemodialysis (23, 24), and its mechanism may be related to factors such as malnutrition, molecular aging, and the use of erythropoiesis-stimulating agents (25, 26). Additionally, we also observed that there was no significant association between the initial total creatinine clearance and total Kt/V in PD patients and their mortality. Further research and discussion are warranted on whether the rapid decline of these indicators is associated with mortality.

While our research highlights the importance of controlling bFBG in PD patients, we acknowledge the challenges that clinicians may face in practical clinical settings. Firstly, factors such as individual variations, underlying conditions, and complications can hinder the application of universal treatment standards to all patients. Secondly, given the unique physiology and metabolic status of PD patients, we recommend thoroughly considering personalized factors when devising treatment strategies. Implementing such personalized treatment approaches may enhance responsiveness to individual patient needs. Lastly, recognizing the complexity of individual differences and treatment responses, it is advisable to maintain flexibility in adjusting the target value of bFBG throughout the treatment process. This adaptability requires close collaboration with patients for timely adjustments based on individual conditions and treatment responses, ultimately facilitating more effective blood sugar control and clinical management.

While our study establishes an association between bFBG levels and mortality rates, it is essential to acknowledge the inherent limitations in inferring causation from observational data. The observed correlation should not be misconstrued as implying a causal relationship. The nature of our investigation, based on observational data, precludes definitive causal claims. It is crucial for readers and practitioners to interpret our findings within the context of association rather than causation, recognizing the complexities involved in establishing direct cause-and-effect relationships in observational studies.

This study has two main implications. Firstly, it reinforces the importance of glycemic control in PD patients, suggesting that both high and low bFBG levels can have negative effects on mortality. Secondly, the identification of a non-linear relationship highlights the need for personalized approaches to managing blood glucose levels, tailoring interventions to the specific needs of each patient.

There are several limitations to this study that should be acknowledged. Firstly, being a retrospective study, it is susceptible to inherent biases and limitations associated with the use of retrospective data. Secondly, the study was conducted at a single center, which may limit the generalizability of the findings. Lastly, the sample size relatively small, which could affect the statistical power of the analysis. We will continue to expand the sample size to further enhance the robustness of our results.

## 5 Conclusion

In summary, our study has demonstrated a non-linear relationship between bFBG and mortality in PD patients. Additionally, we have found that the optimal bFBG value associated with the lowest risk of mortality is approximately 5.1 mmol/L. However, it is important to note that further prospective cohort studies are needed to validate and confirm this relationship between bFBG levels and mortality in patients with PD.

## Data availability statement

The original contributions presented in this study are included in this article/Supplementary materials, further inquiries can be directed to the corresponding author.

## Ethics statement

The studies involving humans were approved by the Affiliated Hospital of Jining Medical University. The studies were conducted in accordance with the local legislation and institutional requirements. Written informed consent for participation was not required from the participants or the participants’ legal guardians/next of kin because of the nature of the retrospective cohort study.

## Author contributions

XL: Conceptualization, Project administration, Writing – original draft, Writing – review & editing. CF: Data curation, Project administration, Writing – review & editing. CW: Writing – review & editing, Data curation. YZ: Writing – review & editing, Methodology, Software. LN: Data curation, Software, Writing – review & editing, Writing – original draft.

## Footnotes

1
http://www.R-project.org
