# Supplementary material for: Non-linear relationship between baseline fasting blood glucose and mortality in peritoneal dialysis patients, a retrospective cohort study
Source: Front Med (Lausanne). 2024 Feb 16;11:1325914. doi: 10.3389/fmed.2024.1325914 (PMC10904652; doi:10.3389/fmed.2024.1325914)
Supplement: Supplementary file 1 [file Table_1.DOCX]

Table S1 Risk factors for mortality in CAPD patients

| Item | Univariate Analysis | |  | Multivariate Analysis | |
| --- | --- | --- | --- | --- | --- |
|  | *HR*(95%CI) | *P* |  | *HR*(95%CI) | *P* |
| FBG | 1.36(1.24,1.49) | <0.001 |  | 1.22(1.05,1.41) | 0.009 |
| Sex,femal | 0.93(0.51,1.7) | 0.807 |  | 0.68(0.31,1.47) | 0.324 |
| Age | 1.08(1.05,1.11) | <0.001 |  | 1.05(1.03,1.08) | <0.001 |
| Diabetes,yes | 5.96(3.31,10.72) | <0.001 |  | 1.71(0.74,3.98) | 0.212 |
| Smoke,yes | 3.25(1.8,5.89) | <0.001 |  | 2.55(1.28,5.09) | 0.008 |
| Hypertension,yes | 0.7(0.35,1.38) | 0.298 |  |  |  |
| WBC(×10^9) | 1.05(0.96,1.16) | 0.266 |  |  |  |
| Hb(g/L) | 1.01(1,1.03) | 0.105 |  |  |  |
| MCV(fL) | 1.05(1,1.11) | 0.074 |  | 1.08(1.02,1.15) | 0.011 |
| PLA(×10^9) | 1.0018(0.9979,1.0057) | 0.377 |  |  |  |
| ALT(U/L) | 0.99(0.98,1) | 0.103 |  |  |  |
| AST(U/L) | 0.9927(0.9753,1.0104) | 0.415 |  |  |  |
| ALP(U/L) | 0.9987(0.9947,1.0027) | 0.52 |  |  |  |
| TP(g/L) | 0.98(0.95,1.02) | 0.31 |  |  |  |
| ALB(g/L) | 0.93(0.89,0.98) | 0.005 |  | 0.99(0.94,1.05) | 0.691 |
| PAB(g/L) | 1.0009(0.9991,1.0026) | 0.348 |  |  |  |
| BUN(mmol/L) | 0.98(0.95,1.01) | 0.121 |  |  |  |
| CR(umol/L) | 0.9982(0.997,1) | 0.005 |  | 0.99(0.99,1) | 0.206 |
| UA(umol/L) | 0.9958(0.993,0.9986) | 0.003 |  | 0.99(0.99,1) | 0.127 |
| cCa(mmol/L) | 3.46(0.82,9.85) | 0.12 |  |  |  |
| P(mmol/L) | 0.61(0.35,1.08) | 0.093 |  | 1.42(0.71,2.81) | 0.321 |
| PTH(pg/mL) | 1.12(1.01,1.23) | 0.101 |  |  |  |
| TC(mmol/L) | 1.12(0.89,1.41) | 0.348 |  |  |  |
| TG(mmol/L) | 1.14(0.84,1.56) | 0.4 |  |  |  |
| HDL(mmol/L) | 1.86(0.87,3.96) | 0.108 |  |  |  |
| LDL(mmol/L) | 1.23(0.87,1.73) | 0.241 |  |  |  |
| WklyCcr(ml/min) | 0.9942(0.9813,1.0072) | 0.382 |  |  |  |
| WklyTotalKt/V | 0.94(0.56,1.57) | 0.806 |  |  |  |
| PDWklyKt/V | 1.0026(0.959,1.0481) | 0.91 |  |  |  |
| RWKt/V | 0.87(0.5,1.52) | 0.63 |  |  |  |
| AHM | 1.34(0.53,3.39) | 0.54 |  |  |  |
| AHD | 0.66(0.36,1.21) | 0.179 |  |  |  |
| Notes: WBC,White Blood Cell Count; Hb, Hemoglobin; PTT, Platelet Count; MCV, Mean Corpuscular Volume; ALT, Alanine Aminotransferase; AST, Aspartate Aminotransferase; ALP, Alkaline Phosphatase; TP, Total Protein; ALB,Albumin; PAB, Prealbumin; BUN,Blood Urea Nitrogen; CR,Creatinine; UA,Uric Acid; cCa, Corrected Calcium; PTH, Phosphorus Parathyroid Hormone; TC, Total Cholesterol; TG, Triglycerides; HDL, High-Density Lipoprotein; LDL, Low-Density Lipoprotein;Wkly Ccr, Weekly creatinine clearance; Wkly Total Kt/V, Weekly Total Kt/V; RW Kt/V, Renal Weekly Kt/V; PD Wkly Kt/V, Peritoneal Dialysis Weekly Kt/V; AHM, Antihyperglycemic Medications; AHD, Antihypertensive drugs. | | | | | |

| Table S2 Table 2 Association between initial bFBG and mortality in diferent models | | | | | | | | | |
| --- | --- | --- | --- | --- | --- | --- | --- | --- | --- |
| Variable | Model1 | | | Model2 | | | Model3 | | |
|  | *HR*^1^ | *95%CI^1^* | *P* | *HR*^1^ | *95%CI^1^* | *P* | *HR*^1^ | *95%CI^1^* | *P* |
| bFBG | 1.36 | 1.24,1.49 | <0.001 | 1.25 | 1.13,1.39 | <0.001 | 1.18 | 1.00,1.40 | 0.049 |
| bFBG |  |  |  |  |  |  |  |  |  |
| Q1 | 1(Ref) |  |  | 1(Ref) |  |  | 1(Ref) |  |  |
| Q2 | 0.62 | 0.2,0.96 | 0.416 | 0.6 | 0.3,0.9 | 0.038 | 0.68 | 0.17,0.87 | 0.037 |
| Q3 | 1.52 | 0.58,3.99 | 0.4 | 1.37 | 0.52,3.63 | 0.523 | 1.98 | 0.57,6.83 | 0.282 |
| Q4 | 3.66 | 1.57,8.55 | 0.003 | 2.39 | 1,3.71 | 0.05 | 1.82 | 1.58,5.72 | 0.038 |
| Trend test | 1.78 | 1.32,2.38 | <0.001 | 1.5 | 1.12,2.01 | 0.007 | 1.35 | 1.35,1.92 | 0.012 |
| ^1^HR=Hazard Ratio,CI=Confidence Interval  Model1 not adjusted.Model 2 adjustedf or Age and Sex.Model3 adjusted for model 2 plus Diabetes, Smoking status, MCV, UA, ALB, Cr, P | | | | | | | | | |
